# Deep learning-based automated high-accuracy location and identification of fresh vertebral compression fractures from spinal radiographs: a multicenter cohort study

**DOI:** 10.3389/fbioe.2024.1397003

**Published:** 2024-05-14

**Authors:** Hao Zhang, Ruixiang Xu, Xiang Guo, Dan Zhou, Tongshuai Xu, Xin Zhong, Meng Kong, Zhimin Zhang, Yan Wang, Xuexiao Ma

**Affiliations:** ^1^ Department of Spinal Surgery, The Affiliated Hospital of Qingdao University, Qingdao, Shandong, China; ^2^ Department of Pain, YanTai YuHuangDing Hospital, Yantai, Shandong, China; ^3^ Department of Spinal Surgery, The Affiliated Hospital of Weifang Medical University, Weifang, Shandong, China; ^4^ Department of Spinal Surgery, Binzhou Medical University Hospital, Binzhou, Shandong, China; ^5^ Department of Radiology, The Affiliated Hospital of Qingdao University, Qingdao, Shandong, China; ^6^ Department of Spinal Surgery, Qingdao Municipal Hospital, Qingdao, Shandong, China; ^7^ Department of Stomatology, The Affiliated Hospital of Qingdao University, Qingdao, Shandong, China

**Keywords:** deep learning, fractures, compression, spine, radiography

## Abstract

**Background:**

Digital radiography (DR) is a common and widely available examination. However, spinal DR cannot detect bone marrow edema, therefore, determining vertebral compression fractures (VCFs), especially fresh VCFs, remains challenging for clinicians.

**Methods:**

We trained, validated, and externally tested the deep residual network (DRN) model that automated the detection and identification of fresh VCFs from spinal DR images. A total of 1,747 participants from five institutions were enrolled in this study and divided into the training cohort, validation cohort and external test cohorts (YHDH and BMUH cohorts). We evaluated the performance of DRN model based on the area under the receiver operating characteristic curve (AUC), feature attention maps, sensitivity, specificity, and accuracy. We compared it with five other deep learning models and validated and tested the model internally and externally and explored whether it remains highly accurate for an external test cohort. In addition, the influence of old VCFs on the performance of the DRN model was assessed.

**Results:**

The AUC was 0.99, 0.89, and 0.88 in the validation, YHDH, and BMUH cohorts, respectively, for the DRN model for detecting and discriminating fresh VCFs. The accuracies were 81.45% and 72.90%, sensitivities were 84.75% and 91.43%, and specificities were 80.25% and 63.89% in the YHDH and BMUH cohorts, respectively. The DRN model generated correct activation on the fresh VCFs and accurate peak responses on the area of the target vertebral body parts and demonstrated better feature representation learning and classification performance. The AUC was 0.90 (95% confidence interval [CI] 0.84–0.95) and 0.84 (95% CI 0.72–0.93) in the non-old VCFs and old VCFs groups, respectively, in the YHDH cohort (*p* = 0.067). The AUC was 0.89 (95% CI 0.84–0.94) and 0.85 (95% CI 0.72–0.95) in the non-old VCFs and old VCFs groups, respectively, in the BMUH cohort (*p* = 0.051).

**Conclusion:**

In present study, we developed the DRN model for automated diagnosis and identification of fresh VCFs from spinal DR images. The DRN model can provide interpretable attention maps to support the excellent prediction results, which is the key that most clinicians care about when using the model to assist decision-making.

## Introduction

Vertebral compression fractures (VCFs) are a common clinical problem, with an annual incidence of approximately 1.5 million in the United States (Goldstein et al., 2015; Madassery, 2020). Osteoporosis, trauma, and neoplastic infiltration are the most common etiological causes of VCFs (Mauch et al., 2018). VCFs are associated with back pain, reduced mobility, and spinal kyphosis (Goldstein et al., 2015). In addition, VCFs may lead to a lower health-related quality of life and higher long-term mortality. Therefore, accurate detection and rapid diagnosis of this condition are essential to ensure that patients with the suspected disease receive timely treatment (Clark et al., 2016; Petritsch et al., 2017; Kim KC. et al., 2021).

Percutaneous vertebroplasty has been widely applied to treat osteoporotic VCFs; however, cement augmentation is only effective for fresh VCFs. Therefore, clinicians must diagnose patients with fresh VCFs as early as possible (differentiating fresh VCFs from normal vertebrae and old VCFs), and promptly evaluate the surgical strategy and etiology of fresh VCFs. Magnetic resonance imaging (MRI), particularly T2-weighted imaging with fat suppression (FS T2WI), is regarded as the most reliable imaging method for determining the presence of a fresh fracture owing to its excellent ability and sensitivity to detect vertebral hemorrhages and bone marrow edema in fresh VCFs (Kaup et al., 2016; Frellesen et al., 2018). However, the long appointment cycle, claustrophobia, and contraindications, such as metal implants, are common limitations of MRI (Chen et al., 2022). Furthermore, the high economic burden and discomfort due to loud noise may prevent its widespread application.

In clinical practice, digital radiography (DR) is the frontline imaging examination for the diagnosis of fractures, including limb and spinal fractures, due to it being the fastest and most accessible imaging modality (Kim KC. et al., 2021; Kim DH. et al., 2021). However, spinal DR primarily provide information about vertebral morphology and cannot detect acute bone marrow edema caused by fresh VCFs; therefore, determining fresh VCFs from spinal DR remains challenging for clinicians. Radiomics and deep learning (DL) models have achieved excellent performance for diagnosis and application (He et al., 2020; Mu et al., 2020). In addition, many DL models based on DR have been developed and used to achieve partial functions of MRI and echocardiography (Jeong et al., 2021).

To the best of our knowledge, a limited number of studies have focused on DL models for identifying fresh VCFs from DR. In this study, we developed the deep residual network (DRN) model that automated the identification of fresh VCFs from spinal DR images and explored whether it remains highly accurate for an external test cohort.

## Materials and methods

### Study design and participants

**FIGURE 1:**
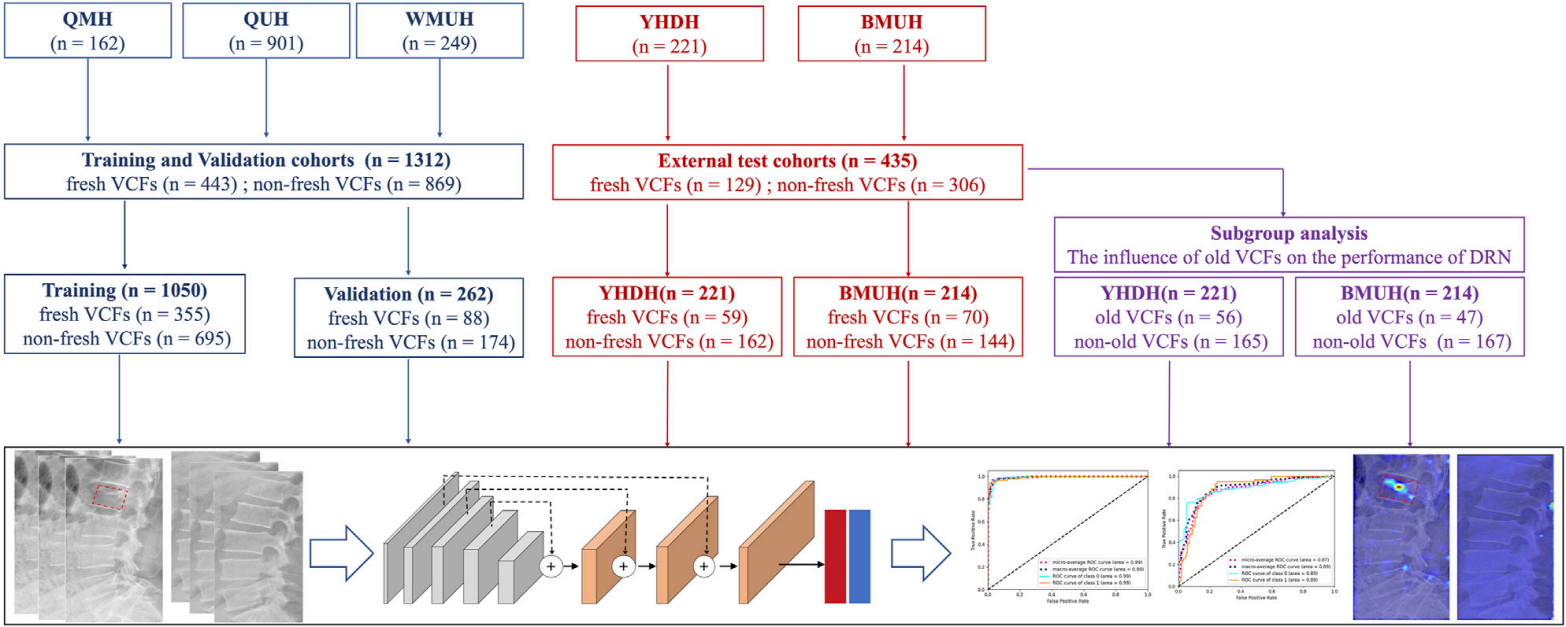
Study flowchart.

This multi-institutional retrospective study included the eligible participants from five institutions. From November 2016 to July 2023, 1,747 participants who underwent spinal MRI and DR within 2 weeks of back pain were enrolled in this study. We excluded 3,858 participants, including those in whom with more than 1 week between DR and MRI examinations (n = 1,745) and those without complete raw data and qualified DR/MRI images (n = 2,113). Notably, participants with old VCFs, those with suspected malignant VCFs, and those who underwent surgical treatments, such as vertebroplasty, fixation, and fusion surgery, were not excluded to improve the robustness of our model. The study flowchart and detailed inclusion and exclusion are shown in Figure 1 and the Supplementary Appendix (p. 3).

This study was approved by the Ethics Committees of the five hospitals and was conducted in accordance with the ethical principles of the Declaration of Helsinki. We used only preexisting medical data; therefore, the requirement for informed consent was waived for this retrospective study. The work was compliant with the STROCSS criteria (Mathew et al., 2021).

### Radiological examination

The participants underwent routine anteroposterior and lateral lumbar spine radiography using DR instruments of six devices. All MR scans were acquired using a 1.5-T or 3.0-T MR system with a multichannel phased-array spine coil, and FS T2WI was performed using a turbo spin-echo sequence. The image acquisition parameters and details of the DR instruments of each institution are presented in the Supplementary Appendix (p. 3). The DR and MR images were stored and retrieved using a picture archiving and communication system.

### Ground truth

The whole spinal DR image, without the segmentation and delineation of region of interest (ROI), were labelled based on spinal MRI. FS T2WI is regarded as the standard reference for identifying fresh VCFs owing to its excellent ability to visualize and characterize high-signal areas and bone marrow edema (Genant and Jergas, 2003; Zhang et al., 2023). The sagittal MRI and lateral DR images were analyzed by at least one radiologist and one spinal surgeon. Any disagreements were resolved through consensus discussions.

### Data partition

A total of 1,312 participants from three institutions were divided into a training cohort (n = 1,050; male: 416, 39.62%) and a validation cohort (n = 262; male: 118, 45.04%) based on the patient identification number (first 80% in the training cohort, the remaining 20% in the validation cohort) to train and validate the DRN model. A total of 221 participants (male: 100, 45.25%) and 214 participants (male: 87, 40.65%) from the other two institutions, were divided into YHDH and BMUH cohorts based on their respective institutions, and were categorized as external test cohorts to evaluate the external generalizability of the DRN model.

### Model development

The DRN model was developed based on a previously reported 101-layer residual network (He et al., 2016), and trained on classified spinal DR images of patients in the training cohort. The architecture of the DRN included an input layer, a first layer (a 7 × 7 convolutional layer and an output channel of 64), and second to fifth layers (four residual modules). Each residual module comprised several residual blocks, a global average pooling layer (performing global average pooling on the output of the last residual module), and a fully connected layer (connecting the output of the global average pooling layer to a fully connected layer for the classification task).

The input images from the training cohort were resized to 224 × 224 pixels. We performed image augmentation and amplification of the original images using a horizontal flip to reduce overfitting. This normalization process allows the image data to converge smoothly during training, thereby improving the model’s stability and training effectiveness. All loss values were summed and used to measure the model’s performance. The DRN model was trained based on ImageNet pre-trained parameters. The Adam optimizer was utilized in this model, and the parameters to be optimized and the learning rate were passed to it. The Adam optimizer is an adaptive learning rate optimization algorithm that automatically adjusts the learning rate based on the gradients of the parameters, which results in more efficient updates of the model’s weights. The learning rate was set to 0.0001. The batch size was 6, and the number of training epochs was 200. All the development processes were performed using the PyTorch framework. Detailed information on the development processes, parameters, software, and packages is presented in the Supplementary Appendix (p. 4).

### Model test and evaluation

We assessed the diagnostic performance of the DRN model in both the validation and external test cohorts using the best model training weights and the same thresholds. In the present study, the DRN model was compared with previous competitive models, including 50-layer residual network (ResNet-50) (He et al., 2016), Shufflenet-v2 (Zhang et al., 2018), EfficientnetV2-S (Tan and Le, 2021), EfficientnetV2-M (Tan and Le, 2021), and EfficientnetV2-L (Tan and Le, 2021) to verify the effectiveness of the DRN model. The diagnostic efficacy of the models for identifying fresh VCFs were analyzed. The true positive (TP), true negative (TN), false positive (FP), false negative (FN), accuracy, balanced accuracy, sensitivity, and specificity of the different models were calculated. The TP event is defined as correct identified the VCFs from X-ray images. Receiver operating characteristic (ROC) curves were used to assess the sensitivity and specificity of the models, and the area under the ROC curves (AUC) was used to describe the discriminative power of the models. In addition, the macro- and micro-averaged ROC curves, precision recall (PR) ROC, and PR AUC were included in the evaluation metrics.

### Subgroup analysis

Both fresh and old VCFs can lead to changes in vertebral morphology. Therefore, in clinical practice, even if there are changes in vertebral morphology observed on a participant’s spinal DR, clinicians still find it challenging to accurately identify fresh VCFs solely through visual inspection. In order to assess the impact of the simultaneous presence of old VCFs on the diagnostic results of the DRN model, we grouped participants of the external test cohorts based on whether they had concomitant old VCFs, and the subgroup analysis was conducted to further evaluate the diagnostic efficiency of the model for fresh VCFs.

### Visualization

Visualization experiments based on gradient-weighted class activation mapping (Grad-CAM) have been previously conducted to locate and identify the activation area over the input images to explain the feature learning and classification results of the DL models (Panwar et al., 2020; Liu C. et al., 2021). In addition, Grad-CAM technology uses gradient information flowing into the last convolution layer to assess the weight of each neuron in the final decision of the fresh VCFs (Mu et al., 2021).

### Statistical analysis

All statistical analyses were performed using SPSS Statistics software version 22.0. (SPSS Inc., Chicago, IL, United States of America). and Python version 3.7. We used the bootstrap sampling to calculate the confidence interval directly (n_bootstrap = 1,000), and take the desired percentile of the bootstrap estimates as the bounds of the confidence interval. Normally distributed continuous variables were represented as means ± standard deviations and categorical variables as percentages (%). The Student’s t-test and Mann–Whitney U test were used to compare continuous clinical variables, where appropriate. The chi-squared test or Fisher’s exact test was used for categorical variables. Statistical significance was set at *p*-value <0.05.

## Results

### Population characteristics

A totally of 1,747 participants (572 fresh VCFs and 1,175 non-fresh VCFs; 721 male and 1,026 female) with a mean age of 62.08 ± 13.92 years, were enrolled in this study (Supplementary Appendix p. 6). The training cohort from the three institutions included 1,050 participants (355 fresh VCFs and 695 non-fresh VCFs; 416 male and 634 female), with a mean age of 62.78 ± 13.50 years. In addition, there were 228 participants diagnosed with lumbar fresh VCFs in the training cohort. The validation cohort from the same three institutions included 262 participants (88 fresh VCFs and 174 non-fresh VCFs; 118 male and 144 female), with a mean age of 61.97 ± 13.05 years, and there were 32 participants diagnosed with lumbar fresh VCFs in the validation cohort. The external test cohorts were obtained from YHDH and BMUH. The YHDH cohort included 221 participants (59 fresh VCFs and 162 non-fresh VCFs; 100 male and 121 female), with a mean age of 56.8 ± 16.26 years. The BMUH cohort included 214 participants (70 fresh VCFs and 144 non-fresh VCFs; 87 male and 127 female), with a mean age of 64.28 ± 13.12 years. Additionally, 117 (11.14%) participants in the training cohort, 31 (11.83%) in the validation cohort, 56 (25.34%) in the YHDH cohort, and 47 (21.96%) in the BMUH cohort were diagnosed with old VCFs (or combined old VCFs). Detailed demographics of each cohort are shown in Table 1 and the Supplementary Appendix (p. 7, 8).

**TABLE 1:** Baseline characteristics of cohorts.

	Training cohort	Validation cohort	External test cohorts	
	n = 1,050	n = 262	YHDH (n = 221)	BMUH (n = 214)
FVCF cases	355 (33.81%)	88 (33.59%)	59 (26.7%)	70 (32.71%)
non-FVCF cases	695 (66.19%)	174 (66.41%)	162 (73.3%)	144 (67.29%)
Age (years)	62.78 ± 13.50	61.97 ± 13.05	56.8 ± 16.26	64.28 ± 13.12
Sex, no. (%)				
Male	416 (39.62%)	118 (45.04%)	100 (45.25%)	87 (40.65%)
Female	634 (60.38%)	144 (54.96%)	121 (54.75%)	127 (59.35%)
FVCF distribution, no. (%)				
Thoracic	127 (35.77%)	56 (63.64%)	22 (37.29%)	23 (32.86%)
Lumbar	228 (64.23%)	32 (36.36%)	37 (62.71%)	47 (67.14%)
Diagnosis of OVCF, no. (%)				
OVCF	117 (11.14%)	31 (11.83%)	56 (25.34%)	47 (21.96%)
non-OVCF	933 (88.86%)	231 (88.17%)	165 (74.66%)	167 (78.04%)

### Visualization experiments

**FIGURE 2:**
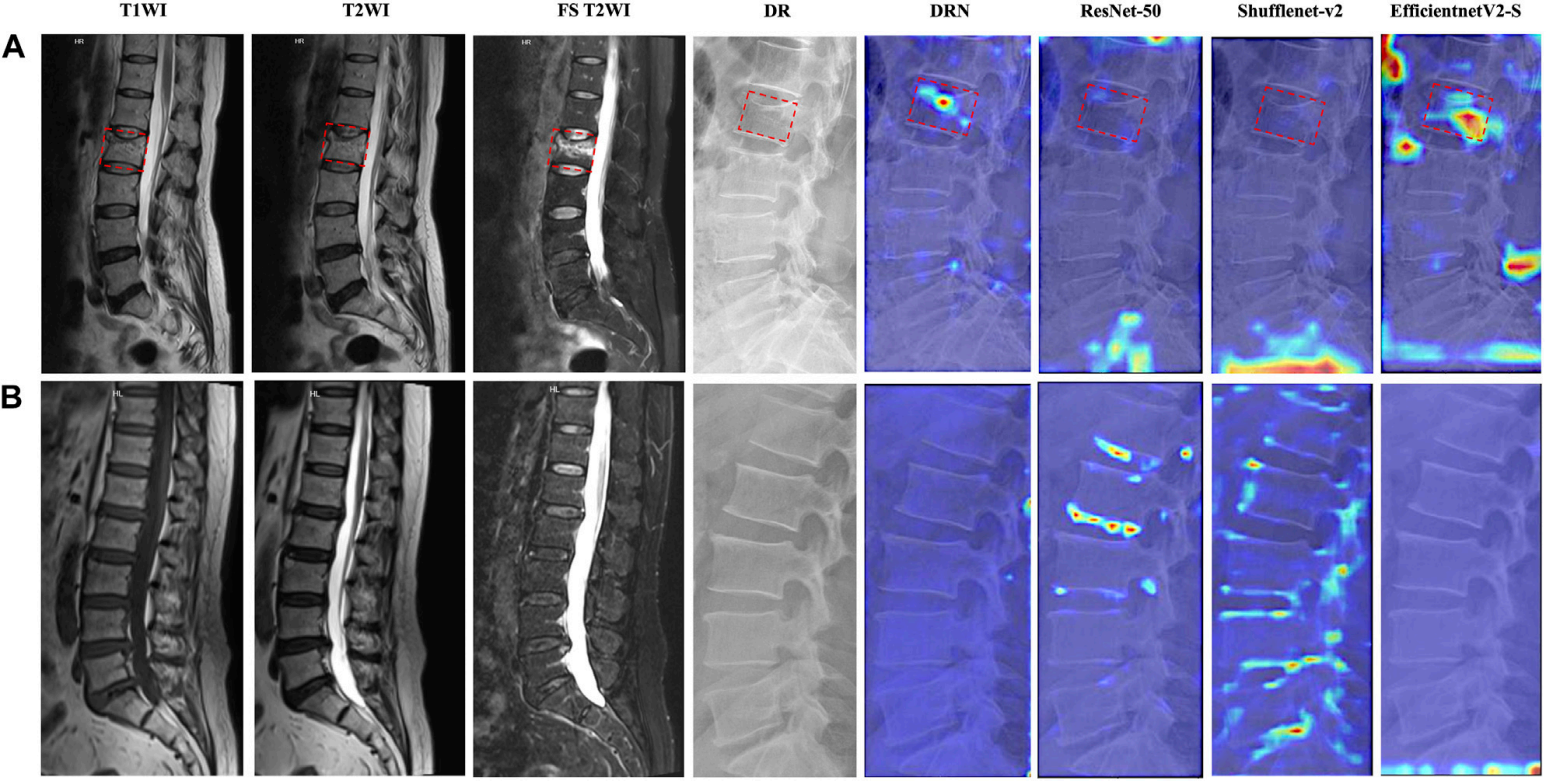
Attention heatmaps of different models for representative participants. T1-weighted imaging (T1WI), T2WI, FS T2WI, DR image, and attention heatmaps of different models of participants who were predicted successfully in **(A)** fresh VCFs and **(B)** non-fresh VCFs group. The fresh VCFs are pointed by the red boxes.

As shown in Figure 2, we visualized the feature attention maps of different DL models using the Grad-CAM technique, which intuitively illustrated the effectiveness of our DRN model. The DRN model generated correct activation on the fresh VCFs and accurate peak responses on the area of the target vertebral body parts and demonstrated better feature representation learning and classification performance. The attention heatmap is designed to refine attention on the most distinctive region; however, ResNet-50, Shufflenet-v2 and EfficientnetV2-S generated wider, even erroneous, activation areas than the target vertebral body.

**FIGURE 3:**
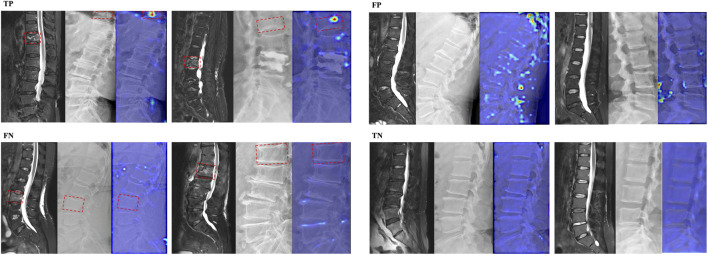
Attention heatmaps of the DRN model in the external test cohorts. The image on the left is the FS T2WI; The image in the middle is corresponding DR image; the image on the right is the corresponding attention heatmap of the DRN model. The fresh VCFs are pointed by the red boxes.

In addition, Grad-CAM demonstrates different feature attention maps for different predicted results of DRN model. The generated focused peak responses for images with FP predictions were similar to those of the TP images; however, the activated area corresponded to the erroneous vertebral body or the area outside of the vertebral body. The DRN model generated a low intensity activation area or no peak responses for TN and FN images (Figure 3).

### Diagnostic performance of the DRN model

**FIGURE 4:**
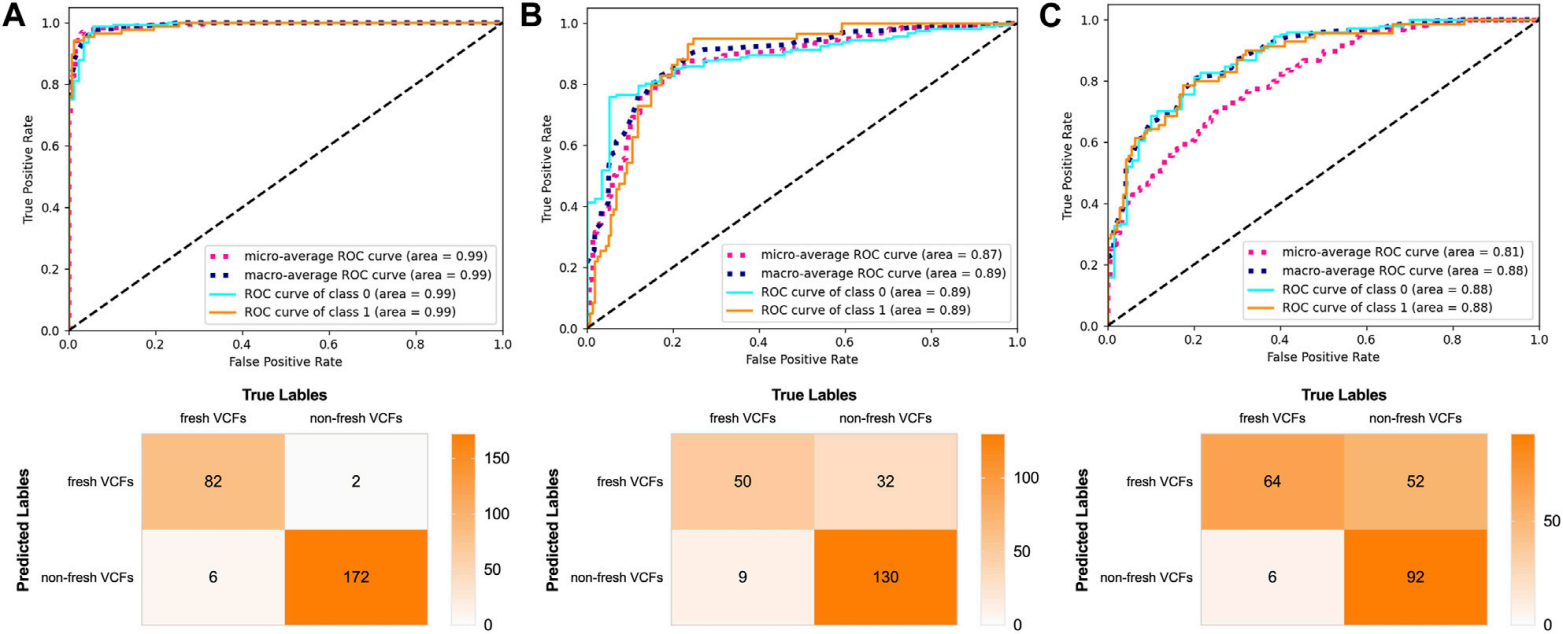
Performance of the DRN model in the validation and external test cohorts. ROC curves and confusion matrix of DRN model in **(A)** the validation cohort, **(B)** the YHDH cohort and **(C)** the BMUH cohort.

The AUC was 0.99, 0.89, and 0.88 in the validation, YHDH, and BMUH cohorts, respectively, for the DRN model for detecting and discriminating fresh VCFs (Figure 4). Moreover, the validation cohort had an accuracy of 96.62%, sensitivity of 93.18%, and specificity of 98.35%. The accuracies were 81.45% and 72.90%, sensitivities were 84.75% and 91.43%, and specificities were 80.25% and 63.89% in the YHDH and BMUH cohorts, respectively (Table 2). The detailed performance of the DRN in different cohorts is shown in the Supplementary Appendix (p. 8, 10). In addition, the results of the DRN with different parameters are shown in the Supplementary Appendix (p. 11, 12).

**TABLE 2:** Performance of the models in the validation and external test cohorts.

	DRN	ResNet-50	Shufflenet-v1	EfficientnetV2-S	EfficientnetV2-M	EfficientnetV2-L
Validation						
Accuracy (%)	96.95	96.95	96.18	72.90	67.56	66.79
Sensitivity (%)	93.18	94.32	95.45	36.36	4.55	2.27
Specificity (%)	98.85	98.28	96.55	91.38	99.43	99.43
Balanced accuracy	96.02	96.30	96.00	63.87	51.99	50.85
YHDH						
Accuracy (%)	81.45	68.33	74.66	59.73	74.66	74.21
Sensitivity (%)	84.75	96.61	91.53	0.00	6.78	6.78
Specificity (%)	80.25	58.02	68.52	81.48	99.38	98.77
Balanced accuracy	82.50	77.32	80.03	40.74	53.08	52.78
BMUH						
Accuracy (%)	72.90	72.90	65.42	54.21	67.75	62.62
Sensitivity (%)	91.43	90.00	91.43	50.00	5.71	11.43
Specificity (%)	63.89	64.58	52.78	56.25	97.92	87.50
Balanced accuracy	77.66	77.29	72.11	53.13	51.82	49.47

**FIGURE 5:**
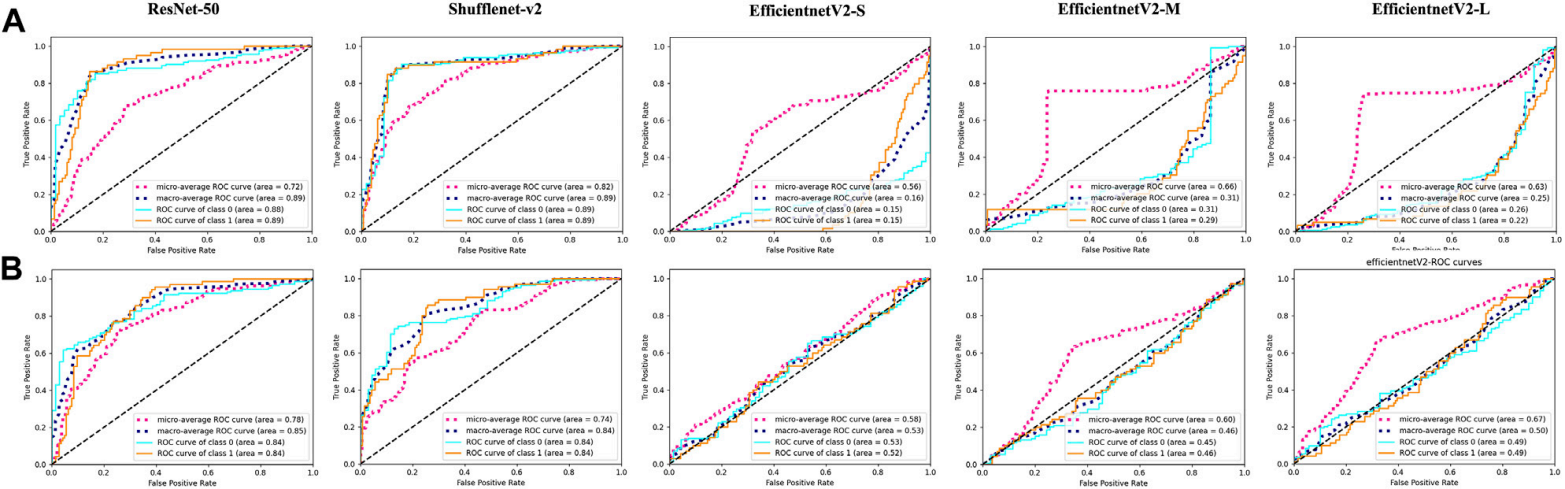
Performance of other models in external test cohorts. ROC curves of ResNet-50, Shufflenet-v2, EfficientnetV2-S, EfficientnetV2-M and EfficientnetV2-L models in **(A)** (the first line) YHDH cohort and **(B)** (the second line) BMUH cohort.

In the present study, the DRN model was compared with previous DL models regarding the ability to discriminate fresh VCFs to evaluate its effectiveness. The AUC of ResNet-50, Shufflenet-v2, EfficientnetV2-S, EfficientnetV2-M, and EfficientnetV2-L were 0.89, 0.89, 0.15, 0.29, and 0.22, respectively, in the YHDH cohort and 0.84, 0.84, 0.52, 0.46, and 0.49, respectively, in the BMUH cohort (Figure 5). The accuracy, sensitivity, and specificity of the validation and external test cohorts for each model are listed in Table 2. The ROC curves in the validation cohort and the overall external test cohort for the models are shown in the Supplementary Appendix (p. 13, 14).

### The influence of old VCFs on the performance of the DRN model

**FIGURE 6:**
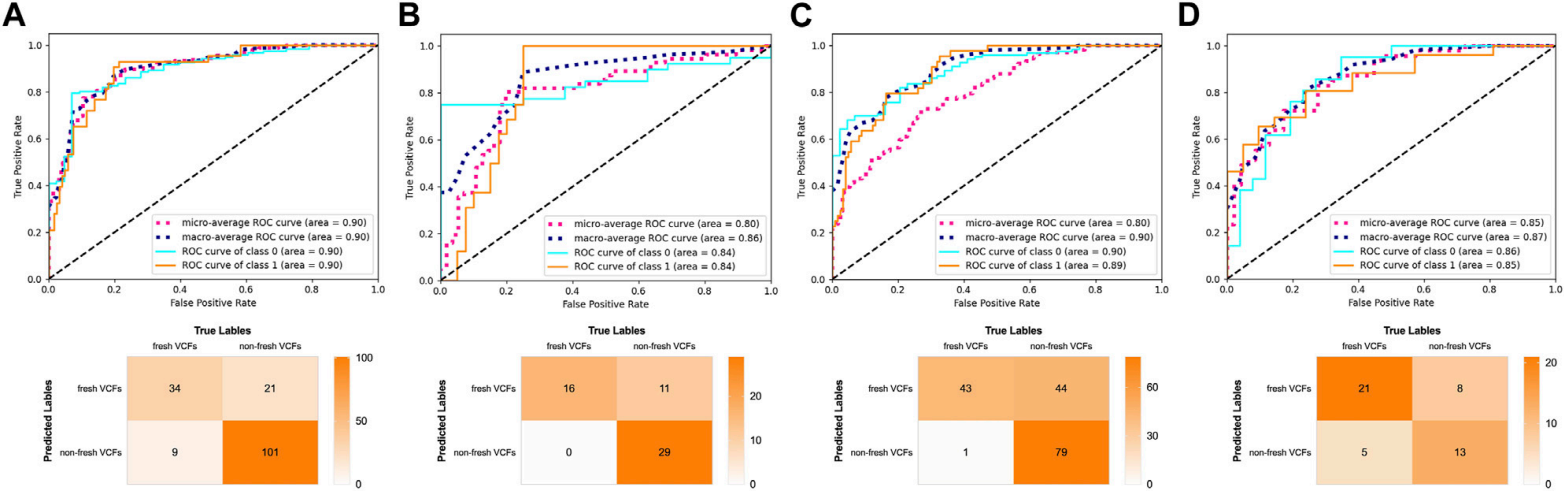
Performance of the DRN model in the subgroups of the external test cohorts. ROC curves and confusion matrix of the DRN model in **(A)** the non-old VCFs group and **(B)** the old VCFs group of the YHDH cohort. ROC curves and confusion matrix of the DRN model in **(C)** the non-old VCFs group and **(D)** the old VCFs group of the BMUH cohort.

**FIGURE 7:**
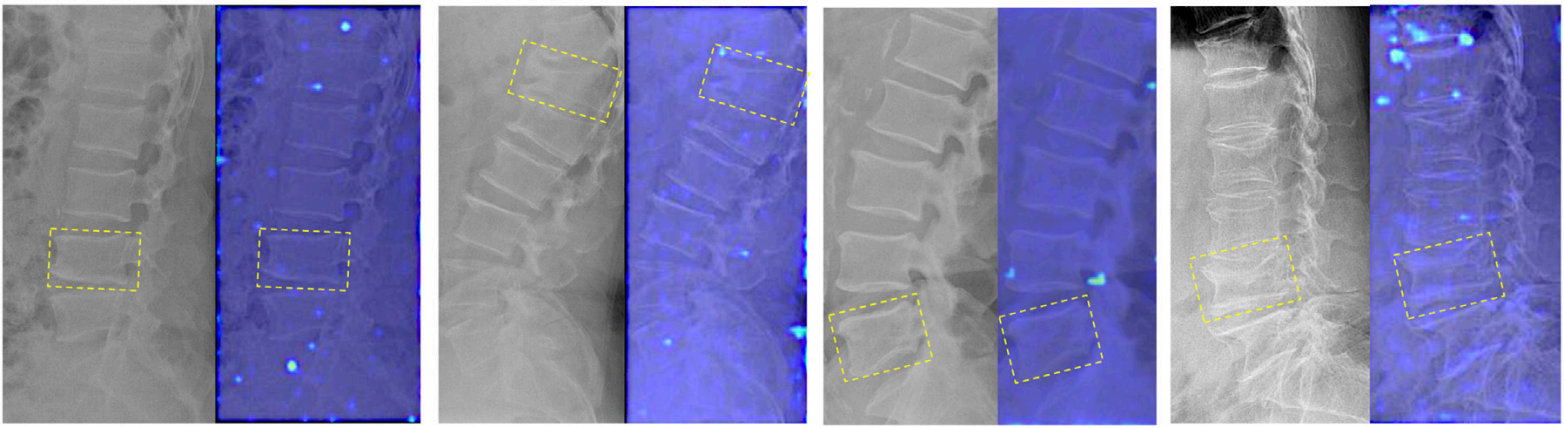
Attention heatmaps of the DRN model in the old VCFs group of the external test cohorts. The image on the left is the DR image of participants without fresh VCFs who were predicted successfully; the image on the right is the corresponding attention heatmap of the DRN model. The old VCFs are pointed by the yellow boxes.

The influence of old VCFs on the performance of the DRN model in identifying fresh VCFs was assessed in the subgroups. Detailed demographics of each subgroup of the YHDH and BMUH cohorts are shown in Table 3. The AUC was 0.90 (95% confidence interval [CI] 0.84–0.95) and 0.84 (95% CI 0.72–0.93) in the non-old VCFs and old VCFs groups, respectively, in the YHDH cohort (*p* = 0.067). The AUC was 0.89 (95% CI 0.84–0.94) and 0.85 (95% CI 0.72–0.95) in the non-old VCFs and old VCFs groups, respectively, in the BMUH cohort (*p* = 0.051) (Table 4). The confusion matrix for each subgroup is shown in Figure 6, and attention heatmaps of DRN in old VCFs group of external test cohorts is shown in Figure 7.

**TABLE 3:** Baseline characteristics of the subgroups in the external test cohorts.

	YHDH cohort			BMUH cohort		
	Non-OVCF (n = 165)	OVCF (n = 56)	P	Non-OVCF (n = 167)	OVCF (n = 47)	P
Age (years)	53.43 ± 16.25	66.74 ± 11.35	<0.001	62.44 ± 12.72	70.81 ± 12.26	<0.001
Sex, no. (%)			0.677			0.331
Male	76 (46.06%)	24 (42.86%)		65 (38.92%)	22 (46.81%)	
Female	89 (53.94%)	32 (57.14%)		102 (61.08%)	25 (53.19%)	
FVCFs, no. (%)			0.714			<0.001
FVCF	43 (26.06%)	16 (28.57%)		44 (26.35%)	26 (55.32%)	
non-FVCF	122 (73.94%)	40 (71.43%)		123 (73.65%)	21 (44.68%)	

**TABLE 4:** Performance of the DRN model for subgroups in the external test cohorts.

	YHDH cohort			BMUH cohort		
	Non-OVCF	OVCF	P	Non-OVCF	OVCF	P
AUC	0.9 (0.84–0.95)	0.84 (0.72–0.93)	0.067	0.89 (0.84–0.94)	0.85 (0.72–0.95)	0.051
Accuracy (%)	81.82 (75.76–87.27)	80.36 (69.64–91.07)	-	73.05 (66.47–79.04)	72.34 (59.57–85.11)	-
Sensitivity (%)	79.07 (66.67–90.01)	100 (100–100)	-	97.73 (92.31–100)	80.77 (65.22–95.83)	-
Specificity (%)	82.79 (75.42–89.08)	72.5 (58.53–85.71)	-	64.23 (55.65–72.36)	61.9 (40.91–82.63)	-
F1 score	0.69 (0.58–0.79)	0.74 (0.56–0.87)	-	0.66 (0.55–0.74)	0.76 (0.63–0.88)	-

## Discussion

MRI is the imaging modality of choice for identifying fresh VCFs; however, its clinical utility is limited by many factors, including the high cost and time involved. DL and machine learning (ML) models have been explored with the development of artificial intelligence and radiomics for the diagnosis and differential diagnosis of fresh VCFs (Chen et al., 2022). Several studies have shown the promising ability of DL and ML models to meet or surpass the performance of human experts on many survival prediction and medical imaging analysis tasks, involving multiple imaging modalities and application areas (Liu X. et al., 2021). However, DL and ML models have not been utilized in large-scale clinical practice. The gap between the success of these models in research and that in clinical settings must be addressed as an essential part of every DL model. At least two major factors hinder the translation of DL models into clinical practice: 1) the workload of manual segmentation, which can hardly be accomplished in a clinical workflow, and 2) the low external generalizability of the model to external institutions. In this study, we successfully overcame this resistance to clinical translation. We developed and evaluated the DRN model for the diagnosis of fresh VCFs from spinal DR images and found that the AUC for the overall model performance was greater than 0.88 in the external test cohorts. The attention heatmaps showed that the DRN model generated the correct activation of the target vertebral body parts and avoided manual segmentation.

### Full automation and visualization

To the best of our knowledge, this study is the first to develop and test a fully automated DL diagnosis model for fresh VCFs based on spinal DR images and achieve visualization. The segmentation and delineation of ROI in DL or ML models require considerable time and effort. Therefore, achieving fully automated positioning and detection of fresh VCFs and reducing the complexity of segmentation is necessary. In previous studies on DL models, the diagnosis and detection of fresh VCFs from spinal DR images required segmentation of each vertebral body from the whole thoracic and lumbar vertebrae (Chen et al., 2022; Xu et al., 2023).

In present study, we achieved fully automated positioning and detected fresh VCFs with no ROI requirement using our DRN model. The local perception property and local feature extraction capability of four convolution layers endows the DRN model with an advantage in handling spinal DR image, enabling the DRN model to capture intricate details and structural information of fresh VCFs, thereby improving the recognition and automatic localization capabilities for the fresh VCFs. Intermediate activation layers were visualized to assess the accuracy of the positioning function in the DL network to explore the detection process and DL features. Our model enables better feature representation learning and a clear activation heatmap compared to other methods, as shown in the results of the Grad-CAM and final output, which allows better automatic positioning performance for the identification of fresh VCFs. In addition, we attempted to identify fresh VCFs in the C-arm DR images based on our models; however, we did not achieve accurate detection. Therefore, multicenter analyses with a large number of C-arm images of the spine are required to explore more stable and accurate DL models to determine the surgical segments and prevent surgical accidents.

In addition, many DL models are dedicated to segmenting bones and vertebrae from radiological images (Huang et al., 2020; Wennmann et al., 2022a). Klinder et al. (Klinder et al., 2009) reported an automated model-based vertebral detection, identification, and segmentation method; this model can achieve an identification success of more than 70% for a single vertebra. Wennmann et al. (Wennmann et al., 2022b) explicitly provided segmentations of vertebrae based on U-Nets, which trained 106 patients from eight centers. These findings may strengthen our future studies on DL models, particularly those focusing on automatic segmentation and the identification of spinal diseases. For example, by utilizing the hierarchical structure of the DRN model as the encoder and integrating the U-shaped architecture of segmentation models like U-Net, we may can achieve a sophisticated approach aimed at enhancing the model’s versatility and performance. This integrated approach not only enhances the model’s versatility, but also opens new possibilities for image segmentation problems in various application scenarios.

### Robustness and external generalizability

Previous studies have reported that bone signal intensities and radiomic features can markedly deviate between different scanners and protocols of MRI, computed tomography, and radiography (Wennmann et al., 2022c; Wennmann et al., 2022d). In addition, models that are developed and tested on a single cohort may be prone to overfitting, resulting in the final model performing well only for images in that cohort and showing low accuracy in other datasets (Ueda et al., 2023).

In previous studies, only a single dataset was used for the development of the DL model, and the images used for development and testing were obtained from the same dataset (Chen et al., 2022; Xu et al., 2023). The training and test cohorts of our study were collected from different institutions and using different scanners to prevent overfitting and enhance robustness and external generalizability. In this study, the participants in the training cohort were recruited from three hospitals, which improved the robustness of the DRN model. In addition, two external test cohorts were used to assess the external generalizability of the DRN model, and a high AUC (0.89 and 0.88 in the YHDH and BMUH cohorts, respectively) was achieved. Although variations in DR instruments, image acquisition parameters, age, gender composition, and other factors may contribute to the model’s slightly superior results in the YHDH cohort, these results still strongly support the stability and excellent performance of our DRN model on external test cohorts.

It is difficult to identify fresh VCFs, particularly differentiating them from old VCFs, based on DR images in clinical practice. Both fresh and old VCFs can result in similar morphological changes in the vertebral body, such as loss of height, wedging, or deformity. These changes can be visually similar, making it difficult to differentiate between the two types VCFs based solely on the limited information of DR images. Therefore, multiple normal vertebral columns and old VCFs were included in this study to improve the robustness of the model and evaluate whether the DRN model’s detection of fresh VCFs would be affected. The results of the subgroup analysis showed that the AUC of the DRN model decreased in the old VCFs group compared to the non-old VCFs group (both in two external test cohorts); however, the *p*-value between the two subgroups was greater than 0.05 (YHDH, *p* = 0.067; BMUH, *p* = 0.051). This indicated that there was no significant difference in the diagnostic performance between the two subgroups, i.e., the diagnostic process of the DRN was not affected by the presence of old VCFs. In addition, as shown in Figure 7, there were no peak responses generated in the area of old VCFs which indicates that the DRN model has learned to distinguish fresh VCFs from normal vertebrae and old VCFs based on specific features.

### Limitations

This study has some limitations. In general, anteroposterior and lateral radiographs are jointly captured for spinal DR interpretation. However, only lateral views are collected and used to identify fresh VCFs in the present study, and the lack of anteroposterior radiographs may have resulted in the loss of some imaging features in the DR images. In addition, the influence of malignant VCFs on the performance of the DRN model and the ability of the DRN model to distinguish between benign and malignant VCFs were not further evaluated. However, noninvasive differentiation of benign *versus* malignant VCFs is a potentially important clinical application, particularly for patients with VCFs without typical morphological features.

## Conclusion

In the present study, we developed the DRN model for automated diagnosis and identification of fresh VCFs from spinal DR images. The DRN model can provide interpretable attention maps to support the excellent prediction results, which is the key that most clinicians care about when using the model to assist decision-making.

## Data Availability

The raw data supporting the conclusion of this article will be made available by the authors, without undue reservation.

## Abbreviations

VCFs, vertebral compression fractures; MRI, magnetic resonance imaging; FS T2WI, T2-weighted imaging with fat suppression; DR, digital radiography; DL, deep learning; DRN, deep residual network; ResNet-50, 50-layer residual network; TP, true positive; TN, true negative; FP, false positive; FN, false negative; ROC, receiver operating characteristic; AUC, the area under the ROC curves; Grad-CAM, gradient-weighted class activation mapping.

## References

[B1] Chen W. Liu X. Li K. Luo Y. Bai S. Wu J. (2022). A deep-learning model for identifying fresh vertebral compression fractures on digital radiography. Eur. Radiol. 32 (3), 1496–1505. 10.1007/s00330-021-08247-4 34553256

[B2] Clark W. Bird P. Gonski P. Diamond T. H. Smerdely P. McNeil H. P. (2016). Safety and efficacy of vertebroplasty for acute painful osteoporotic fractures (vapour): a multicentre, randomised, double-blind, placebo-controlled trial. Lancet (London, Engl.) 388 (10052), 1408–1416. 10.1016/s0140-6736(16)31341-1 27544377

[B3] Frellesen C. Azadegan M. Martin S. S. Otani K. D'Angelo T. Booz C. (2018). Dual-energy computed tomography-based display of bone marrow edema in incidental vertebral compression fractures: diagnostic accuracy and characterization in oncological patients undergoing routine staging computed tomography. Investig. Radiol. 53 (7), 409–416. 10.1097/rli.0000000000000458 29489560

[B4] Genant H. K. Jergas M. (2003). Assessment of prevalent and incident vertebral fractures in osteoporosis research. Osteoporos. Int. 14 (Suppl. 3), S43–S55. 10.1007/s00198-002-1348-1 12730798

[B5] Goldstein C. L. Chutkan N. B. Choma T. J. Orr R. D. (2015). Management of the elderly with vertebral compression fractures. Neurosurgery 77 (Suppl. 4), S33–S45. 10.1227/neu.0000000000000947 26378356

[B6] He B. Dong D. She Y. Zhou C. Fang M. Zhu Y. (2020). Predicting response to immunotherapy in advanced non-small-cell lung cancer using tumor mutational burden radiomic biomarker. J. Immunother. Cancer 8 (2), e000550. 10.1136/jitc-2020-000550 32636239 PMC7342823

[B7] He K. Zhang X. Ren S. Sun J. (2016). “Deep residual learning for image recognition,” in Paper presented at: Conference on Computer Vision and Pattern Recognition (CVPR), Las Vegas, NV, USA, 27-30 June 2016 (IEEE), 770–778.

[B8] Huang J. Shen H. Wu J. Hu X. Zhu Z. Lv X. (2020). Spine explorer: a deep learning based fully automated program for efficient and reliable quantifications of the vertebrae and discs on sagittal lumbar spine mr images. Spine J. 20 (4), 590–599. 10.1016/j.spinee.2019.11.010 31759132

[B9] Jeong H. G. Kim B. J. Kim T. Kang J. Kim J. Y. Kim J. (2021). Classification of cardioembolic stroke based on a deep neural network using chest radiographs. EBioMedicine 69, 103466. 10.1016/j.ebiom.2021.103466 34229276 PMC8264106

[B10] Kaup M. Wichmann J. L. Scholtz J. E. Beeres M. Kromen W. Albrecht M. H. (2016). Dual-energy ct-based display of bone marrow edema in osteoporotic vertebral compression fractures: impact on diagnostic accuracy of radiologists with varying levels of experience in correlation to mr imaging. Radiology 280 (2), 510–519. 10.1148/radiol.2016150472 26928067

[B11] Kim D. H. Jeong J. G. Kim Y. J. Kim K. G. Jeon J. Y. (2021b). Automated vertebral segmentation and measurement of vertebral compression ratio based on deep learning in x-ray images. J. digital imaging 34 (4), 853–861. 10.1007/s10278-021-00471-0 PMC8455797 34236562

[B12] Kim K. C. Cho H. C. Jang T. J. Choi J. M. Seo J. K. (2021a). Automatic detection and segmentation of lumbar vertebrae from x-ray images for compression fracture evaluation. Comput. methods programs Biomed. 200, 105833. 10.1016/j.cmpb.2020.105833 33250283

[B13] Klinder T. Ostermann J. Ehm M. Franz A. Kneser R. Lorenz C. (2009). Automated model-based vertebra detection, identification, and segmentation in ct images. Med. Image Anal. 13 (3), 471–482. 10.1016/j.media.2009.02.004 19285910

[B14] Liu C. Xie H. Zhang Y. (2021a). Self-supervised attention mechanism for pediatric bone age assessment with efficient weak annotation. IEEE Trans. Med. Imaging 40 (10), 2685–2697. 10.1109/tmi.2020.3046672 33351757

[B15] Liu X. Zhang D. Liu Z. Li Z. Xie P. Sun K. (2021b). Deep learning radiomics-based prediction of distant metastasis in patients with locally advanced rectal cancer after neoadjuvant chemoradiotherapy: a multicentre study. EBioMedicine 69, 103442. 10.1016/j.ebiom.2021.103442 34157487 PMC8237293

[B16] Madassery S. (2020). Vertebral compression fractures: evaluation and management. Seminars interventional radiology 37 (2), 214–219. 10.1055/s-0040-1709208 PMC7224975 32419735

[B17] Mathew G. Agha R. Albrecht J. Goel P. Mukherjee I. Pai P. (2021). Strocss 2021: strengthening the reporting of cohort, cross-sectional and case-control studies in surgery. Int. J. Surg. 96, 106165. 10.1016/j.ijsu.2021.106165 34774726

[B18] Mauch J. T. Carr C. M. Cloft H. Diehn F. E. (2018). Review of the imaging features of benign osteoporotic and malignant vertebral compression fractures. AJNR Am. J. Neuroradiol. 39 (9), 1584–1592. 10.3174/ajnr.A5528 29348133 PMC7655272

[B19] Mu W. Jiang L. Shi Y. Tunali I. Gray J. E. Katsoulakis E. (2021). Non-invasive measurement of pd-l1 status and prediction of immunotherapy response using deep learning of pet/ct images. J. Immunother. Cancer 9 (6), e002118. 10.1136/jitc-2020-002118 34135101 PMC8211060

[B20] Mu W. Jiang L. Zhang J. Shi Y. Gray J. E. Tunali I. (2020). Non-invasive decision support for nsclc treatment using pet/ct radiomics. Nat. Commun. 11 (1), 5228. 10.1038/s41467-020-19116-x 33067442 PMC7567795

[B21] Panwar H. Gupta P. K. Siddiqui M. K. Morales-Menendez R. Bhardwaj P. Singh V. (2020). A deep learning and grad-cam based color visualization approach for fast detection of covid-19 cases using chest x-ray and ct-scan images. Chaos Solit. Fractals 140, 110190. 10.1016/j.chaos.2020.110190 PMC7413068 32836918

[B22] Petritsch B. Kosmala A. Weng A. M. Krauss B. Heidemeier A. Wagner R. (2017). Vertebral compression fractures: third-generation dual-energy ct for detection of bone marrow edema at visual and quantitative analyses. Radiology 284 (1), 161–168. 10.1148/radiol.2017162165 28240561

[B23] Tan M. Le Q. (2021). “Efficientnetv2: smaller models and faster training,” in Paper presented at: International conference on machine learning.

[B24] Ueda D. Matsumoto T. Ehara S. Yamamoto A. Walston S. L. Ito A. (2023). Artificial intelligence-based model to classify cardiac functions from chest radiographs: a multi-institutional, retrospective model development and validation study. Lancet Digit. Health 5 (8), e525–e533. 10.1016/s2589-7500(23)00107-3 37422342

[B25] Wennmann M. Bauer F. Klein A. Chmelik J. Grözinger M. Rotkopf L. T. (2022d). *In vivo* repeatability and multiscanner reproducibility of mri radiomics features in patients with monoclonal plasma cell disorders: a prospective bi-institutional study. Invest. Radiol. 58, 253–264. 10.1097/rli.0000000000000927 36165988

[B26] Wennmann M. Klein A. Bauer F. Chmelik J. Grözinger M. Uhlenbrock C. (2022b). Combining deep learning and radiomics for automated, objective, comprehensive bone marrow characterization from whole-body mri: a multicentric feasibility study. Invest. Radiol. 57 (11), 752–763. 10.1097/rli.0000000000000891 35640004

[B27] Wennmann M. Neher P. Stanczyk N. Kahl K. C. Kächele J. Weru V. (2022a). Deep learning for automatic bone marrow apparent diffusion coefficient measurements from whole-body magnetic resonance imaging in patients with multiple myeloma: a retrospective multicenter study. Invest. Radiol. 58, 273–282. 10.1097/rli.0000000000000932 36256790

[B28] Wennmann M. Thierjung H. Bauer F. Weru V. Hielscher T. Grözinger M. (2022c). Repeatability and reproducibility of adc measurements and mri signal intensity measurements of bone marrow in monoclonal plasma cell disorders: a prospective bi-institutional multiscanner, multiprotocol study. Invest. Radiol. 57 (4), 272–281. 10.1097/rli.0000000000000838 34839306

[B29] Xu F. Xiong Y. Ye G. Liang Y. Guo W. Deng Q. (2023). Deep learning-based artificial intelligence model for classification of vertebral compression fractures: a multicenter diagnostic study. Front. Endocrinol. (Lausanne) 14, 1025749. 10.3389/fendo.2023.1025749 37033240 PMC10073698

[B30] Zhang H. Yuan G. Wang C. Zhao H. Zhu K. Guo J. (2023). Differentiation of benign versus malignant indistinguishable vertebral compression fractures by different machine learning with mri-based radiomic features. Eur. Radiol. 33 (7), 5069–5076. 10.1007/s00330-023-09678-x 37099176

[B31] Zhang X. Zhou X. Lin M. Sun J. (2018). “Shufflenet: an extremely efficient convolutional neural network for mobile devices,” in Paper presented at: Proceedings of the IEEE conference on computer vision and pattern recognition (IEEE), 6848–6856.

