# Supplementary material for: Deep learning-based automated high-accuracy location and identification of fresh vertebral compression fractures from spinal radiographs: a multicenter cohort study
Source: Front Bioeng Biotechnol. 2024 May 14;12:1397003. doi: 10.3389/fbioe.2024.1397003 (PMC11135169; doi:10.3389/fbioe.2024.1397003)
Supplement: Supplementary file 1 [file DataSheet1.PDF]

Supplementary appendix

Deep learning-based automated high-accuracy location and identification of fresh vertebral compression fractures from spinal radiographs: a multicenter cohort study

Table of Contents:

Section S1: Supplementary Methods

1) The detailed inclusion and exclusion criteria.

2) Image acquisition parameters and the details of DR instruments for each institutions.

3) Detailed information of development processes, parameter, software, packages of the deep learning models.

Section S2: Supplementary Tables

Supplementary Table 1: Detail baseline characteristics of all participants.

Supplementary Table 2: Detail baseline characteristics of each cohort.

Supplementary Table 3: Detail performance of the DRN model.

Supplementary Table 4: Baseline characteristics of overall external test cohort.

Supplementary Table 5: Performance of DRN in over external test cohort.

Supplementary Table 6: Difference of DRN performance according to initial weight.

Supplementary Table 7: Difference of DRN performance according to optimizer.

Section S3: Supplementary Figures

Supplementary Figure 1: ROC in validation cohort for models.

Supplementary Figure 2: ROC in overall external test cohort for each model.

Supplementary Figure 3: PR AUC of DRN in each cohorts.

Section S4: References for the Supplementary Material

Section S1: Supplementary Methods

1) The detailed inclusion and exclusion criteria

The inclusion criteria were as follows:

(1) participants within 2 weeks of back pain and underwent spinal MR and DR.

(2) normal participants; or participants with fresh VCFs (trauma or osteoporosis).

The exclusion criteria were as follows:

(1) more than 1 week between DR and MRI examinations (n = 1745).

(2) participants without complete raw data and qualified DR/MRI images (n = 2113).

Finally, a total of 3858 participants were excluded from the study. Of note, to improve the robustness of our model, participants with old VCFs, participants with suspected malignant VCFs (n = 13) and participants who underwent surgical treatment such as vertebroplasty (n = 19; 13 single segment, 5 double segments and 1 three segments), fixation and fusion surgery (n =1; L3-L5) were not excluded in our study.

2) Image acquisition parameters and the details of DR and MRI instruments for each institutions

The participants from QUH were mainly using: a Digital Diagnost (Philips) and a DRX-EVOLUTION PIUS (Carestream), the voltages of the lumbar anteroposterior and lateral films were 75-85 kV, and the currents were 25-75 Ams; a Siemens Skyra 3.0-T MRI with a spine array coil. Sagittal T2WI with dixon was performed using a turbo spin-echo sequence (TR = 2600 ms, TE = 110 ms), the FOV was 310*310; a Philips Achieva 1.5T MRI with a spine array coil. Sagittal T2WI with STIR was performed using a short time inversion recovery (TR = 2600 ms, TE = 100 ms,TI=165ms).

The participants from QMH were mainly using: a DRX-EVOLUTION PIUS (Carestream), the voltages of the lumbar anteroposterior and lateral films were 75-85 kV, and the currents were 15-20 Ams; a Philips Achieva 1.5-T MRI with a spine array coil. Sagittal T2WI with FS was performed using a turbo spin-echo sequence (TR = 3313 ms, TE = 90 ms).

The participants from WMUH were using: a Digital Diagnost (CARESTREAM DirectView System), the voltages of the lumbar anteroposterior and lateral films were 78-85 kV, and the currents were 200-220 Ams; a Philips Prodiva 1.5-T MRI with a spine array coil. Sagittal T2WI with FS was perfjieormed using a turbo spin-echo sequence (TR=3259 ms，TE=100 ms,;TR=2500 ms，TE=60 ms); a GE Signa 3.0-T MRI with a spine array coil. Sagittal T2WI with FS was performed using a turbo spin-echo sequence (TR=2300 ms，TE=125 ms,;TR=2460 ms，TE=125 ms), the FOV was 280*280.

The participants from YHDH were mainly using: a Digital Diagnost (Philips), the voltages of the lumbar anteroposterior and lateral films were 77-90 kV, and the currents were 33-38 Ams; a Philips Ingenia CX 3.0T with a spine array coil. Sagittal T2WI with FS was performed using a turbo spin-echo sequence (TR = 3100 ms, TE = 95 ms).

The participants from BMUH were using: a Yiso (SIEMENS), the voltages of the lumbar anteroposterior and lateral films were 75-85 kV, and the currents were 35-50 Ams; a Siemens Magnetom Avanto 1.5-T MRI (Siemens AG) with a spine array coil. Sagittal T2WI with FS was performed using a turbo spin-echo sequence (TR = 4000 ms, TE = 100 ms), the FOV was 310*310.

3) Detailed information of development processes, parameter, software, packages of the deep learning models

In this study, we determined the most suitable parameters for our model through the following steps. As for the model, we examined DRN, ResNet-50,[1] Shufflenet-v1,[2] EfficientnetV2-S,[3] EfficientnetV2-M[3] and EfficientnetV2-L.[3] Hyperparameter tuning for the optimizer, learning rate, initial weight, image size, and batch size was performed. For the optimizer, we evaluated the stochastic gradient descent (SGD) and Adam; for the initial weight, we evaluated the influence of initial weight of ImageNet, and the best weight was choose from training epochs (epochs = 200); for the learning rate, the searching range for stochastic gradient descent was 0.0001 and default parameters were used for the other optimizers; for the image size, the searching range was 128, 244, 256, and 512 pixels; for batch size, the searching range was 2–32. The best performing

model was DRN. The best optimizer was Adam (learning rate 0.0001), initial weight was used, and batch size was 6. For the validation and test set, each image was resized to 256 pixels, cropped centrally to 224 pixels, and passed through the developed model. Logit values outputted from the model were transformed by softmax function into probabilities.

The software liberties and packages we used included python 3.7, opencv-python 4.8.0, pytorch 3.7.1, matplotlib 3.5.3, numpy 1.21.5, pandas 1.3.5, scipy 1.7.3, torchvision 0.10.0, and scikit-learn 1.0.2

Section S2: Supplementary Tables

Supplementary Table 1: Detail baseline characteristics of all participants

	ALL participants (n=1747)
**Fresh VCFs cases**	572 (32.74%)
**Non-fresh VCFs cases**	1175 (67.26%)
**Age (years)**	62.08±13.92
**Sex, no. (%)**	
Male	721 (41.27%)
Female	1026 (58.73%)
**Fresh VCFs distribution, no. (%)**	
Thoracic	228 (39.86%)
Lumbar	344 (60.14%)
**Diagnosis of old VCFs, no. (%)**	
Old VCFs	235 (13.45%)
Non-old VCFs	1496 (85.63%)
**Ethnic group, no. (%)**	
Chinese	1747 (100%)
Other	0 (0%)

Data are n (%) or mean (SD).

Supplementary Table 2: Detail baseline characteristics of each cohort

	Training cohort (n=1050)			Validation cohort (n=262)			YHDH cohort (n=221)			BMUH cohort (n=214)		
	Fresh VCFs (n=355)	Non-fresh VCFs (n=695)	P	Fresh VCFs (n=88)	Non-fresh VCFs (n=174)	P	Fresh VCFs (n=59)	Non-fresh VCFs (n=162)	P	Fresh VCFs (n=70)	Non-fresh VCFs (n=144)	P
**Age (years)**	70.71±11.88	58.72±12.44	<0.001	69.97±11.43	57.93±11.93	<0.001	66.31±10.98	53.34±16.51	<0.001	72.26±9.76	60.4±12.80	<0.001
**Sex, no. (%)**			<0.001			<0.001			<0.001			0.055
Male	108(30.42%)	308(44.32%)		26(29.55%)	92(52.87%)		9(15.25%)	91(56.17%)		22(31.43%)	65(45.14%)	
Female	247(69.58%)	387(55.68%)		62(70.45%)	82(47.13%)		50(84.75%)	71(43.83%)		48(68.57%)	79(54.86%)	
**Segmental distribution of fresh VCFs, no. (%)**												
Thoracic	127(35.77%)	-		56(63.64%)	-		22(37.29%)	-		23(32.86%)	-	
Lumbar	228(64.23%)	-		32(36.36%)	-		37(62.71%)	-		47(67.14%)	-	
**Suspected malignant VCFs, no. (%)**	12 (3.38%)	-	-	1 (1.14%)	-		0 (0%)	-		0 (0%)	-	
**History of spinal surgery, no. (%)**												
Vertebroplasty	10 (2.82%)	1 (0.14%)		5 (5.68%)	0 (0%)		0 (0%)	0 (0%)		3 (4.28%)	0 (0%)	
Fixation and fusion surgery	0 (0%)	0 (0%)		0 (0%)	0 (0%)		1 (1.69%)	0 (0%)		0 (0%)	0 (0%)	

Data are n (%) or mean (SD).

Supplementary Table 3: Detail performance of the DRN model

	AUC (95% CI)	Accuracy (%, 95% CI)	Sensitivity (%, 95% CI)	Specificity (%, 95% CI)	F1 score (95% CI)
**Validation cohort**	0.99(0.98-1.00)	96.62(94.66-98.85)	93.18(87.23-97.78)	98.85(97.06-1.00)	0.95(91.85-98.04)
**YHDH cohort**	0.89(0.84-0.92)	81.45(76.01-86.43)	84.75(75.44-92.86)	80.25(73.58-85.80)	0.71(61.95-78.57)
**BMUH cohort**	0.88(0.82-0.92)	72.9(66.82-78.97)	91.43(84.51-97.33)	63.89(56.16-71.03)	0.69(0.60-0.77)

Supplementary Table 4: Baseline characteristics of overall external test cohort

	Overall external test cohort (n=435)		
	**Fresh VCFs (n=129)**	**Non-fresh VCFs (n=306)**	**P**
**Age (years)**	69.53±10.72	56.66±15.27	<0.001
**Sex, no. (%)**			<0.001
Male	31(24.03%)	156(50.98%)	
Female	98(75.97%)	150(49.02%)	
**Segmental distribution of fresh VCFs, no. (%)**			
Thoracic	45(34.88%)	-	
Lumbar	84(65.12%)	-	

Data are n (%) or mean (SD).

Supplementary Table 5: Performance of DRN in over external test cohort

	AUC (95% CI)	Accuracy (%, 95% CI)	Sensitivity (%, 95% CI)	Specificity (%, 95% CI)	F1 score (95% CI)	TP	TN	FN	FP
**DRN**	0.88 (0.84-0.91)	77.24 (73.10-80.70)	88.37 (82.46-93.55)	72.55 (67.65-77.33)	0.70 (0.64-0.75)	114	222	15	84

Supplementary Table 6: Difference of DRN performance according to initial weight

	Initial weight	non-Initial weight
**Validation cohort**		
AUC (95% CI)	0.99(0.98-1.00)	0.96(0.93-0.98)
Accuracy (%, 95% CI)	96.62(94.66-98.85)	91.22(87.79-94.66)
Sensitivity (%, 95% CI)	93.18(87.23-97.78)	81.82(73.68-89.66)
Specificity (%, 95% CI)	98.85(97.06-1.00)	95.98(92.93-98.75)
F1 score (95% CI)	0.95(91.85-98.04)	0.86(0.81-0.91)
**YHDH cohort**		
AUC (95% CI)	0.89(0.84-0.92)	0.82(0.75-0.88)
Accuracy (%, 95% CI)	81.45(76.01-86.43)	83.26(77.83-87.78)
Sensitivity (%, 95% CI)	84.75(75.44-92.86)	57.63(44.44-69.70)
Specificity (%, 95% CI)	80.25(73.58-85.80)	92.59(88.67-96.51)
F1 score (95% CI)	0.71(61.95-78.57)	0.65(0.53-0.75)
**BMUH cohort**		
AUC (95% CI)	0.88(0.82-0.92)	0.49(0.41-0.57)
Accuracy (%, 95% CI)	72.9(66.82-78.97)	44.86(38.32-51.87)
Sensitivity (%, 95% CI)	91.43(84.51-97.33)	72.86(62.12-82.54)
Specificity (%, 95% CI)	63.89(56.16-71.03)	31.25(24.00-38.97)
F1 score (95% CI)	0.69(0.60-0.77)	0.46(0.38-0.54)

Supplementary Table 7: Difference of DRN performance according to optimizer

	Adam	SGD
**Validation cohort**		
AUC (95% CI)	0.99(0.98-1.00)	0.67(0.70-0.75)
Accuracy (%, 95% CI)	96.62(94.66-98.85)	69.85(64.49-75.57)
Sensitivity (%, 95% CI)	93.18(87.23-97.78)	11.36(5.00-18.18)
Specificity (%, 95% CI)	98.85(97.06-1.00)	99.43(98.21-100)
F1 score (95% CI)	0.95(91.85-98.04)	20.2(9.21-30.48)
**YHDH cohort**		
AUC (95% CI)	0.89(0.84-0.92)	0.62(0.53-0.72)
Accuracy (%, 95% CI)	81.45(76.01-86.43)	76.92(71.93-82.36)
Sensitivity (%, 95% CI)	84.75(75.44-92.86)	27.12(16.07-39.66)
Specificity (%, 95% CI)	80.25(73.58-85.80)	95.06(91.77-98.16)
F1 score (95% CI)	0.71(61.95-78.57)	0.39(0.25-0.51)
**BMUH cohort**		
AUC (95% CI)	0.88(0.82-0.92)	0.48(0.40-0.56)
Accuracy (%, 95% CI)	72.9(66.82-78.97)	64.49(57.94-70.56)
Sensitivity (%, 95% CI)	91.43(84.51-97.33)	2.86(0.00-7.36)
Specificity (%, 95% CI)	63.89(56.16-71.03)	94.44(90.65-97.87)
F1 score (95% CI)	0.69(0.60-0.77)	0.05(-)

Section S3: Supplementary Figures

Supplementary Figure 1: ROC curves in validation cohort for models

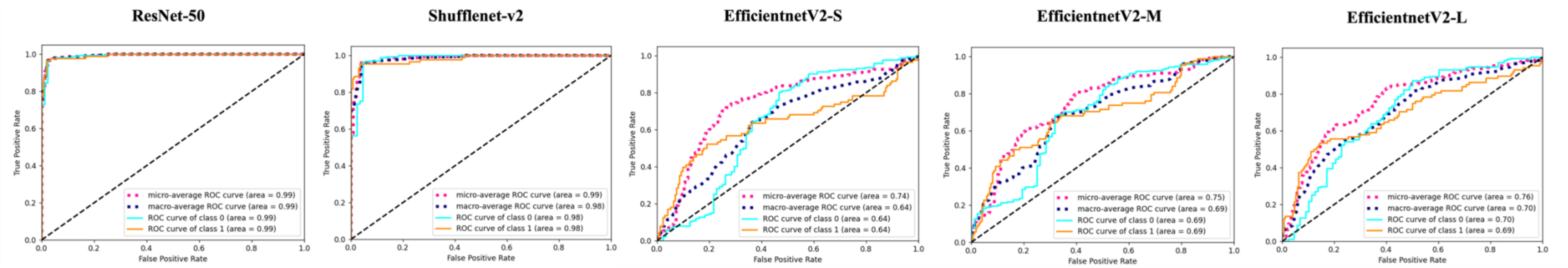

Supplementary Figure 2: ROC curves in overall external test cohort for each model

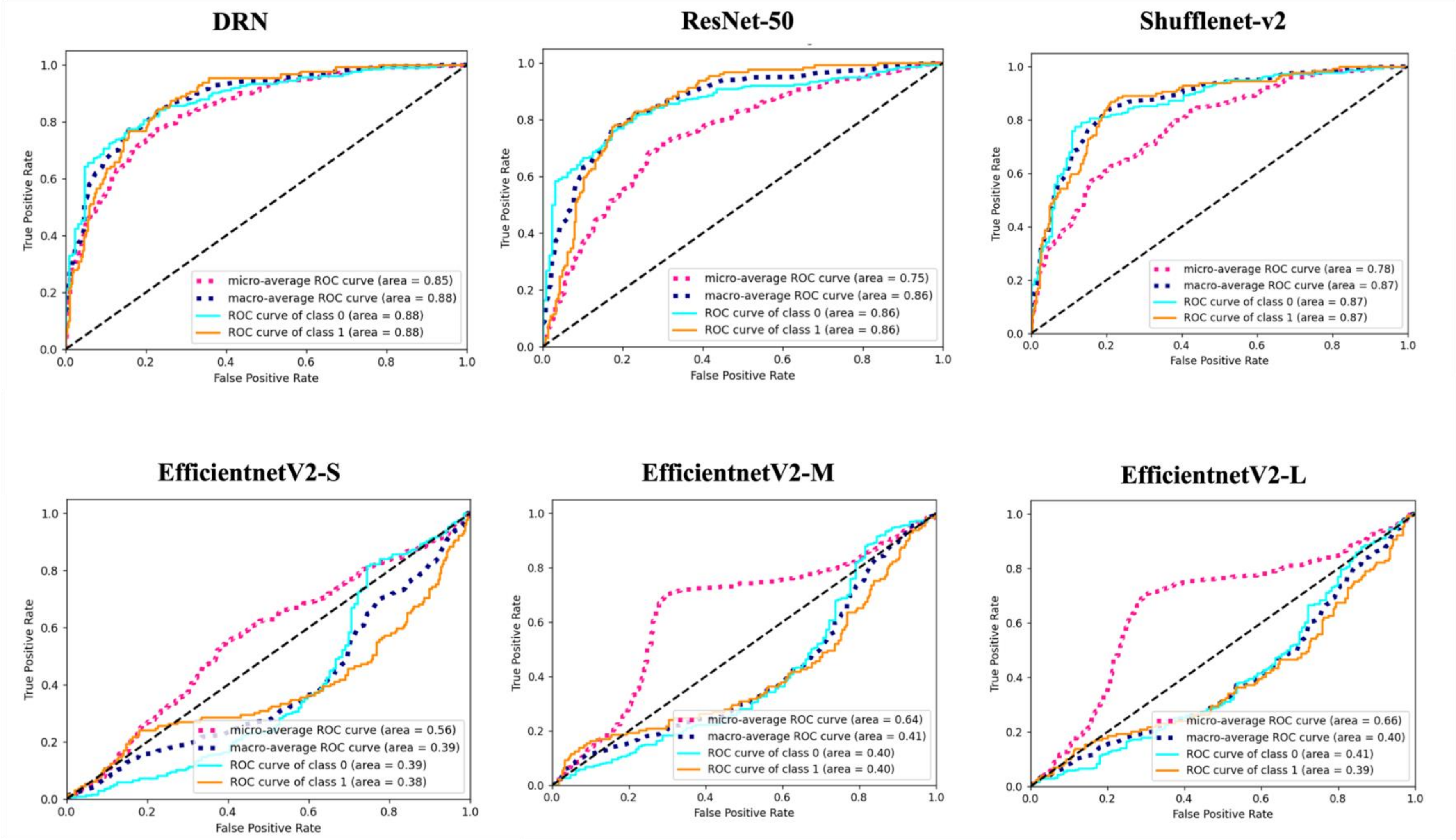

Supplementary Figure 3: PR AUC of DRN in each cohorts (red line: the reference line of random classifier in each dataset**)**

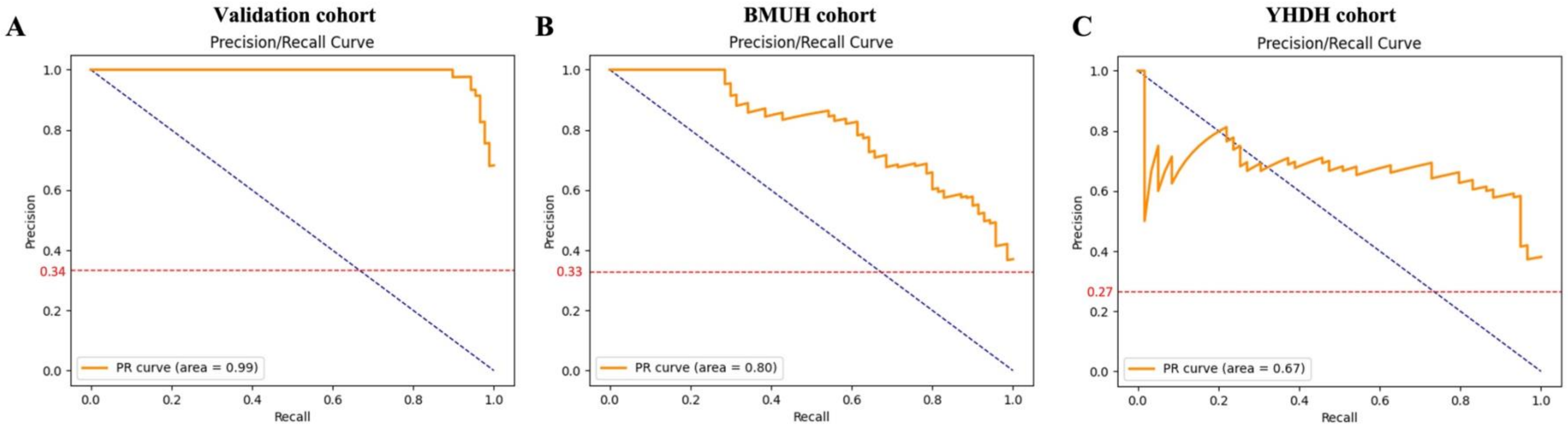

Section S4: References for the Supplementary Material

1. He K, Zhang X, Ren S, Sun J. Deep residual learning for image recognition. Conference on Computer Vision and Pattern Recognition (CVPR). 2016.
2. Zhang X, Zhou X, Lin M, Sun J. Shufflenet: An extremely efficient convolutional neural network for mobile devices. Proceedings of the IEEE conference on computer vision and pattern recognition. 2018:6848-6856.
3. Tan M, Le Q. Efficientnetv2: Smaller models and faster training. International conference on machine learning. 2021:10096-10106.
